# Orchard variable-rate spraying method integrating GNSS and wind-excited audio-conducted leaf area density

**DOI:** 10.3389/fpls.2025.1621080

**Published:** 2025-07-18

**Authors:** Hangxing Zhao, Shenghui Yang, Wenwei Li, Han Feng, Shijie Jiang, Weihong Liu, Jingbin Li, Yongjun Zheng, Songchao Zhang

**Affiliations:** ^1^ Key Laboratory of Modern Agricultural Equipment, Ministry of Agriculture and Rural Affairs, Nanjing, China; ^2^ College of Engineering, China Agricultural University, Beijing, China; ^3^ State Key Laboratory of Intelligent Agricultural Power Equipment, Beijing, China; ^4^ College of Agricultural Equipment Engineering, Henan University of Science and Technology, Luoyang, China; ^5^ College of Mechanical and Electrical Engineering, Shihezi University, Shihezi, China

**Keywords:** GNSS, variable-rate spraying, leaf area density, prescription map, canopy droplet deposition

## Abstract

**Introduction:**

Conventional air-assisted sprayers used in orchards often suffer from excessive pesticide waste, high residue levels, and uneven droplet distribution on fruit tree canopies. Precision spraying technologies have emerged to address these limitations by enabling dynamic regulation of spray parameters according to canopy characteristics. Among these, leaf area density is a key indicator for describing canopy sparseness. However, accurate and automated measurement of canopy leaf area density remains challenging due to leaf shading effects. As a result, few fully functional variable-rate spraying systems have been developed based on this parameter.

**Methods:**

This study presents a variable-rate spraying method that integrates global navigation satellite system (GNSS) positioning with wind-excited audio-conducted estimation of canopy leaf area density. A self-propelled orchard spraying platform was developed to acquire real-time GNSS positioning and audio-conducted canopy leaf area density data. Based on this, a method was established for generating prescription maps that integrate spatial positioning and canopy density information. A variable-rate spray control model and algorithm were then constructed to regulate spray flow according to the spatial distribution of leaf area density across the orchard.

**Results:**

Field experiments demonstrated that the system achieved a mean relative error of only 5.52% in spray flow rate regulation. Compared with conventional constant-rate spraying, the variable-rate mode reduced the longitudinal coefficient of variation (CV) of droplet deposition by 55.75% on adaxial leaf surfaces and by 33.22% on abaxial surfaces, with a maximum reduction of 62.32% in transverse CV. Ground runoff of spray solution was reduced by 62.29%, and droplet deposition density on leaf surfaces exceeded 25 droplets/cm², meeting the standard for low-volume insecticide application.

**Discussion:**

The integration of GNSS and wind-excited audio sensing for real-time canopy density assessment enables more precise and efficient pesticide application in orchards. This system significantly improves droplet deposition uniformity while reducing environmental losses, offering a promising technical solution for the development of intelligent and sustainable plant protection equipment.

## 1 Introduction

Efficient control of orchard pests and diseases is essential for ensuring high fruit yield and quality (Jiang et al., 2022a; Hu et al., 2025). Chemical spraying remains the predominant method for plant protection due to its high efficiency (Rincón et al., 2020; Zheng et al., 2020; Jiang et al., 2022b). Air-assisted spraying, recognized by the Food and Agriculture Organization (FAO) of the United Nations as an advanced plant protection technology, significantly enhances pesticide utilization efficiency and reduces spray drift (Gu et al., 2022a; Yang et al., 2022; Feng et al., 2024). However, conventional uniform spraying methods apply the same spray dosage across all fruit trees based solely on preset parameters. These approaches fail to consider spatial variations in canopy structure and leaf area density (Planas et al., 2013; Colaço et al., 2019a, b).

As a result, they often lead to localized over-application and untreated zones, which not only waste pesticides and increase environmental pollution but also cause inconsistent pest control outcomes (Zhai et al., 2018; Li et al., 2024a). With the growing adoption of precision agriculture, variable-rate spraying (VRS) has emerged as a preferred solution for efficient and environmentally friendly orchard operations (Song et al., 2020). VRS systems integrate sensing, decision-making, and actuation to enable spatially differentiated spraying based on canopy variability. Such systems significantly improve pesticide use efficiency, reduce environmental impact, and optimize the balance between operational efficiency and pest control uniformity (Xue et al., 2023).

Accurate sensing of fruit tree canopies is fundamental to the effectiveness of variable-rate spraying systems (He X., 2020). Among various canopy characteristics, leaf area density is a key indicator of canopy sparsity and directly influences the spatial allocation of spray volume (Wei et al., 2022). Existing methods for leaf area density detection mainly include ultrasonic sensing (Gil et al., 2007; Palleja and Landers, 2015), light detection and ranging (LiDAR) (Hosoi and Omasa, 2007; Li et al., 2017), and multispectral or RGB imaging (Tu et al., 2019; Raj et al., 2021). Ultrasonic sensors estimate canopy volume or LEAF AREA DENSITY by emitting acoustic pulses and measuring their echoes (Palleja and Landers, 2017). However, they are highly susceptible to environmental noise. Even minor distance fluctuations can result in volume estimation errors up to 50 mm, with relative deviations reaching 30% (Palleja et al., 2010). LiDAR acquires three-dimensional point clouds of the canopy by emitting laser pulses, enabling precise reconstruction of canopy structure and estimation of leaf area index (LAI) and volume distribution (Gu et al., 2022b). Nevertheless, in regions where multiple leaf layers overlap and the interlayer spacing is smaller than the divergence angle of the laser beam, LiDAR often fails to penetrate dense canopies. It can only capture the surface geometry, lacking the ability to provide insights into internal leaf area density (Wang and Fang, 2020). Multispectral and RGB imaging estimate LEAF AREA DENSITY using vegetation indices (e.g., NDVI, GNDVI) or reconstruct canopy models through deep learning combined with stereo vision (Liu et al., 2021a). However, their accuracy is significantly compromised by fluctuations in lighting, background interference, and occlusion from overlapping foliage (Wang et al., 2024).Conventional sensors, such as LiDAR and multispectral cameras, therefore fall short of meeting the requirements for internal canopy sensing under field conditions (Li et al., 2024b). To address these limitations, novel sensing strategies must be explored to improve the accuracy and robustness of LEAF AREA DENSITY estimation. Audio-conducted sensing has recently demonstrated promising potential in agricultural applications (Cao et al., 2025). Our research group has observed that the amplitude of wind-induced canopy audio signals correlates with LEAF AREA DENSITY (Li et al., 2024b). This suggests that canopy vibration sounds induced by wind can indirectly reflect internal LEAF AREA DENSITY. (Li et al., 2024c). Unlike optical or laser-based methods, audio signals are inherently immune to lighting fluctuations and leaf occlusion. By extracting time–frequency features from wind-excited canopy audio, it becomes feasible to achieve leaf area density classification even under complex field noise conditions.

The decision-making and actuation mechanisms of variable-rate spraying systems are generally categorized into two types: sensor-based real-time variable-rate spraying systems and prescription map-based variable-rate spraying systems (Zhou et al., 2017). Sensor-based variable-rate spraying systems typically utilize a single type of sensor to detect canopy characteristics such as position, volume, and structural features in real time (Zhang et al., 2018; Gu et al., 2020; Luo et al., 2024). Spray valves are regulated at the millisecond level through pulse-width modulation (PWM) technology to achieve immediate response. However, in complex orchard environments, sensor-based systems are highly susceptible to environmental factors such as lighting conditions, wind speed, and leaf occlusion, which constrain both detection accuracy and decision-making speed (Whitfield et al., 2022). Moreover, supporting high-frequency data decoding and dynamic canopy feature computation requires each sprayer to be equipped with high-performance onboard computing and sensing devices. This significantly increases system investment costs and maintenance expenses (Han et al., 2023). In contrast, prescription map-based variable-rate spraying systems pre-generate spatially referenced prescription maps using multi-source sensor data (Román et al., 2021). During operation, RTK-GPS technology is employed to resolve the corresponding prescription units in real time (Tayari et al., 2015; Liu et al., 2024; Taseer and Han, 2024). In spraying execution, the system only needs to adjust the duty cycle of the solenoid valves via PWM according to the spray rates specified in the prescription map (Partel et al., 2021; Zhang et al., 2024). This approach significantly reduces the hardware requirements for online computation and sensing. It also enables the integration of multiple decision-making factors—such as terrain, canopy structure, and historical growth information—through multi-source data fusion (Tewari et al., 2020). As a result, prescription map-based systems achieve both high spray response speed and high crop information accuracy. Consequently, they exhibit greater application potential and broader promotional value in orchard scenarios (Yu et al., 2022).

In summary, to address the challenges in sensing costs, system integration, and adaptability in variable-rate spraying research, this study proposes an orchard variable-rate spraying system based on wind-induced audio recognition. The main contributions are as follows:

1. Design of a compact self-propelled variable-rate spraying platform: A small-scale, self-propelled spraying platform was developed. It is capable of acquiring orchard-wide positioning data via GNSS and capturing canopy leaf area density using wind-excited audio signals under field conditions.
2. Development of a prescription map generation method integrating canopy leaf area density and spatial positioning information: A mapping model was established to link leaf area density, required spray volume, and PWM duty cycle. Based on this model, a grid-based prescription map was generated by integrating GNSS-based spatial positioning and classified leaf area density levels, providing spatial guidance for precision spraying.
3. Establishment and integration of a variable-rate spraying control model: A control model and algorithm utilizing orchard-wide leaf area density distribution were formulated, enabling dynamic adjustment of spraying rates according to canopy structural variability. These developments led to the full integration of a complete variable-rate spraying system.

Through these innovations and implementations, this study aims to provide a cost-effective and dynamically adaptive LiDAR solution for smart orchards.

## 2 Materials and methods

### 2.1 Design of a compact self-propelled variable-rate spraying platform

**Figure 1:**
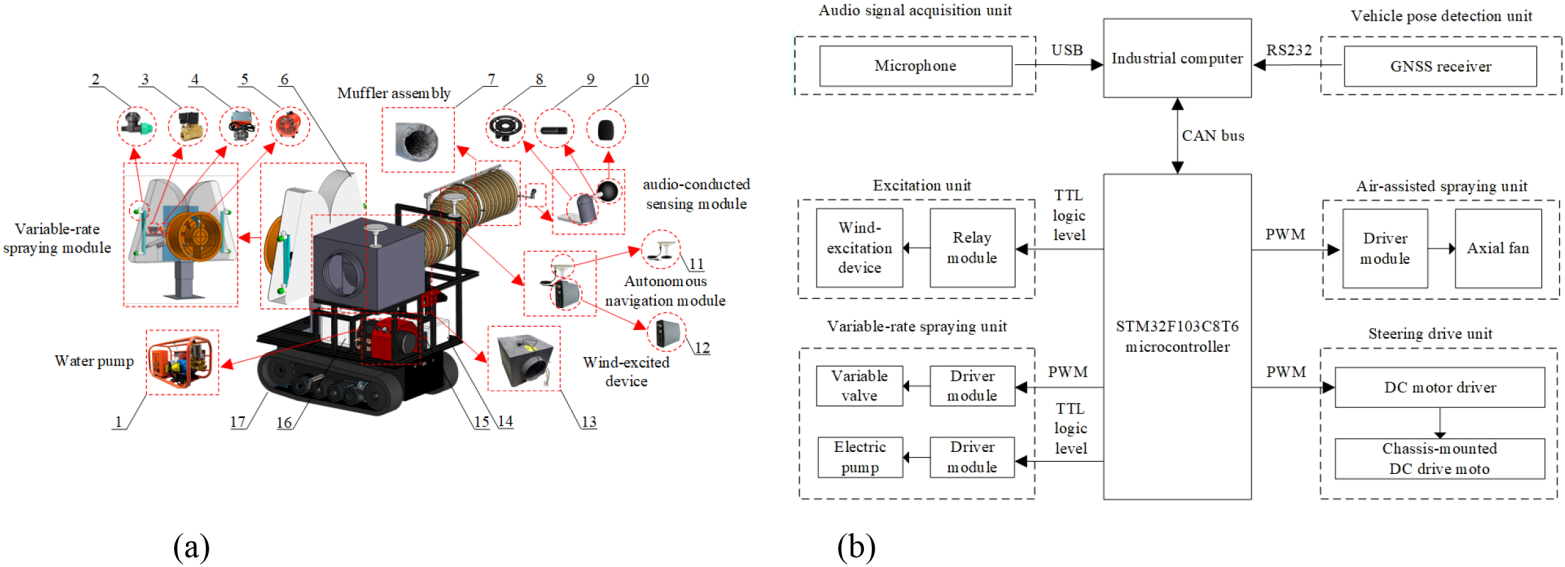
Self-propelled small-scale variable-rate orchard sprayer. 1. Triplex plunger pump 2. Spray nozzle 3. Relay 4. Variable valve 5. Axial fan 6. Air chamber 7. Muffler duct 8. Anti-vibration frame 9. PM420 microphone 10. Windproof sponge 11. GNSS antenna 12. P3-DU receiver 13. wind-excited device 14. Industrial compute 15. Inverter 16. Chemical tank 17. Electric tracked chassis. **(a)** Overall structural diagram **(b)** Hardware block diagram of the control system.

The compact self-propelled variable-rate sprayer developed in this study (**Figure 1a**; **Table 1**) employs a modular integrated architecture driven by the Robot Operating System (ROS). The platform integrates an air-assisted spraying system, a variable-rate spray module, a wind-excited audio-conducted sensing module, an RTK-GNSS-based autonomous navigation system, and an electric tracked chassis. The air-assisted system consists of a high-flow, high-pressure axial-flow fan, an air chamber, and internal guide vanes. Based on the principle of flow displacement, the system generates a uniform dynamic pressure field within the canopy, ensuring efficient droplet penetration and uniform deposition. The spraying module incorporates a three-cylinder plunger pump with a recirculation function, an electric ball valve, and Teejet TP6503 flat-fan nozzles. Spray flow rates are precisely regulated by adjusting the PWM duty cycle applied to the valve. The wind-excited excitation unit is composed of a DPT30-80M centrifugal fan and multilayer vibration isolation and silencing structures. Adjustable wind speeds are used to stimulate branch and leaf vibrations. The audio-conducted sensing module is equipped with a PM420 microphone fitted with a windscreen and an anti-vibration mount. It enables real-time transmission of audio signals for canopy leaf area density estimation. The navigation module utilizes a Huace P3-DU dual-antenna RTK-GNSS receiver. After universal transverse mercator (UTM) projection, operation trajectories are smoothed using the least squares method (Liu et al., 2021b). Steering commands are generated in real time based on the pure pursuit algorithm (Hu et al., 2024), achieving centimeter-level path tracking and closed-loop control for variable-rate spraying.

**Table 1:** Main technical specifications of the compact self-propelled orchard variable-rate sprayer.

Parameter	Value	Remarks
Dimensions (L × W × H) (mm × mm × mm)	1575×1190×1465	Height adjustable
Minimum turning radius (m)	0	
Operating speed (m·s⁻¹)	0~1	
Operating width (m)	≥3	
Fan air volume (m³·h⁻¹)	14900	
Tank Capacity (L)	45	
Pump flow rate (L·min⁻¹)	0~22	Flow adjustable
Pump pressure (MPa)	0~4.0	Pressure adjustable
P3-DU receiver positioning accuracy	Horizontal: 1.0 cm + 1 ppm Vertical: 1.5 cm + 1 ppm	
Heading accuracy (°)	<0.2	

The hardware block diagram of the control system is shown in **Figure 1b**. The upper computer, a Zhanmei GK7000 industrial computer running under the ROS framework, integrates RTK-GNSS positioning, vehicle pose estimation, and audio data acquisition. These functions are used for prescription map generation and path planning. The lower computer, based on an STM32F103C8T6 microcontroller, performs closed-loop control of the excitation device, variable-rate valve, fan, and steering actuators via the CAN bus. The system supports both remote-control and autonomous operation modes.

### 2.2 Prescription map generation method integrating canopy leaf area density and spatial positioning information

#### 2.2.1 Wind-excited audio signal acquisition and leaf area density classification

**Figure 2:**
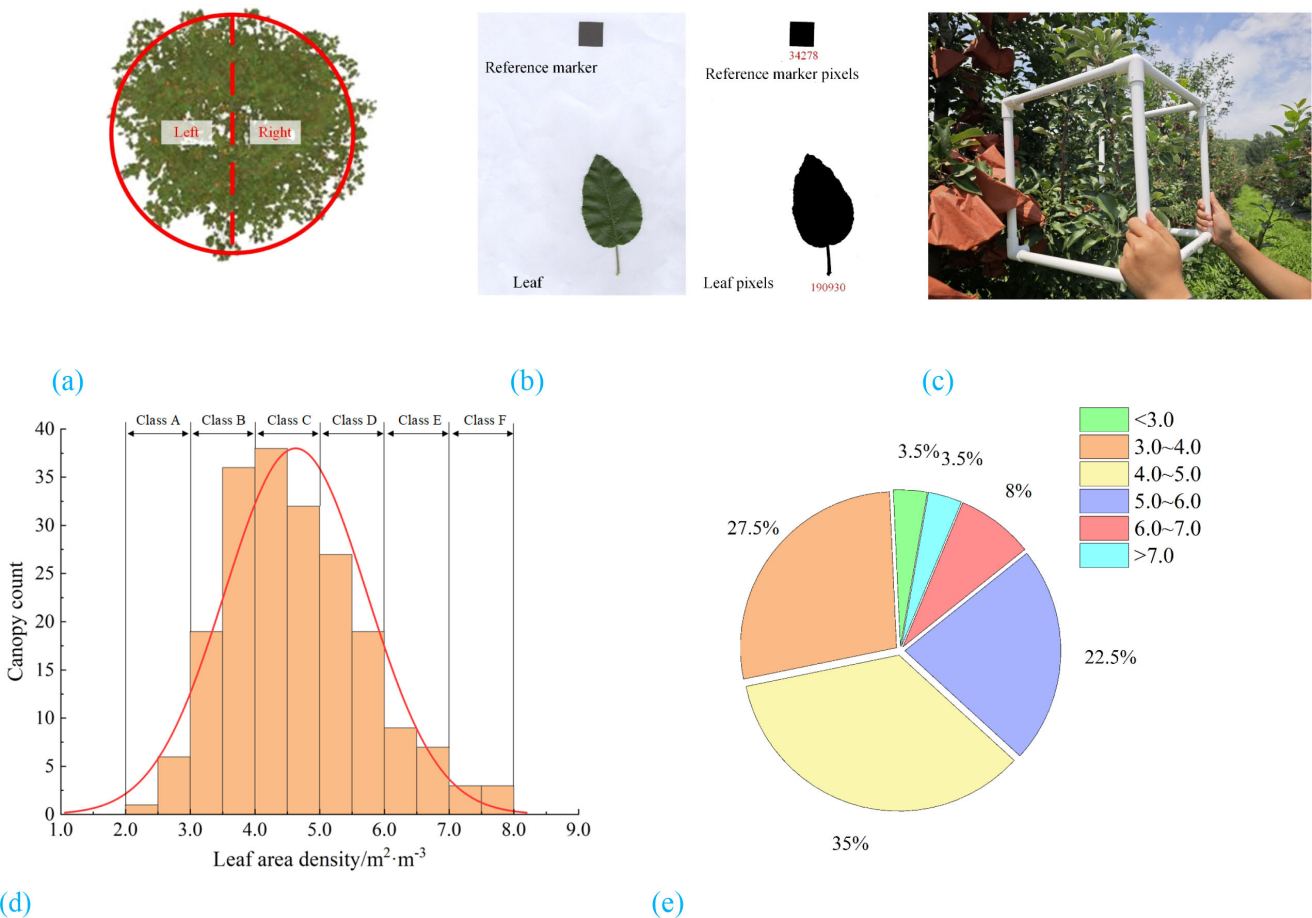
Canopy leaf area density distribution. **(a)** Canopy sampling area **(b)** Leaf area calculation **(c)** Leaf count measurement **(d)** Distribution of canopy counts by leaf area density **(e)** Proportion of canopy counts by leaf area density.

A preliminary field survey was carried out by randomly selecting 100 apple trees to assess canopy leaf area density. In the preliminary experiments, the leaf area density measurement procedure is illustrated in **Figures 2a–c** and includes the following steps:

##### 2.2.1.1 Selection and labeling of target canopy sections

A total of 100 apple trees were randomly selected from the demonstration orchard. Each tree canopy was divided into left and right sections (**Figure 2a**), resulting in 200 canopy regions in total. Each region was labeled numerically (1–200) to facilitate subsequent acquisition of leaf area density and wind-induced audio signals.

##### 2.2.1.2 Leaf area density measurement of the tree canopy

As shown in **Figure 2b**, 50 leaves were randomly sampled from each selected tree. The average leaf area was calculated using Equation 1. Starting from region 1, a PVC square frame was used to delimit the canopy area to be measured (**Figure 2c**). The number of leaves within the frame was manually counted. This procedure was repeated three times at different locations within the same canopy region to obtain an average leaf count. Finally, the leaf area density of the region was calculated using Equation 2.


(1) $$S_{leaf}=\frac{1}{n_{\text{l}}}\sum_{i=1}^{n_{\text{l}}}\frac{N_{leaf,i}\cdot S_{ref,i}}{N_{ref,i}}$$

where *S_leaf_* is the average area of a single leaf (m²); *n_l_* is the number of randomly selected leaves; *S_ref,i_* is the area of the reference black square (4 × 10⁻^4^ m²); *N_leaf,i_* is the number of pixels corresponding to the leaf; and *N_ref,i_* is the number of pixels corresponding to the reference black square.


(2) $$\rho_{leaf}=\frac{N_{c}\cdot S_{leaf}}{V_{c}}$$

where *ρ_leaf_* is the canopy leaf area density (m²·m⁻³); *Nc* is the number of leaves within the canopy; and *Vc* is the canopy volume (m³).

The results showed that the canopy leaf area density ranged from 2.0 m²·m⁻³ to 8.0 m²·m⁻³, with 85.5% of the samples falling within the range of 3.0 m²·m⁻³ to 6.0 m²·m⁻³ (**Figures 2d, e**). Due to the minimal differences in the required spray volume between adjacent density intervals, the leaf area density was classified into six levels (A–F) based on increments of 1.0 m²·m⁻³. This classification approach eliminates the need for precise numerical estimation and allows variable-rate spraying decisions to be made directly according to the assigned category. Consequently, it reduces model complexity and ensures the efficiency and accuracy of spraying decision-making.

**Figure 3:**
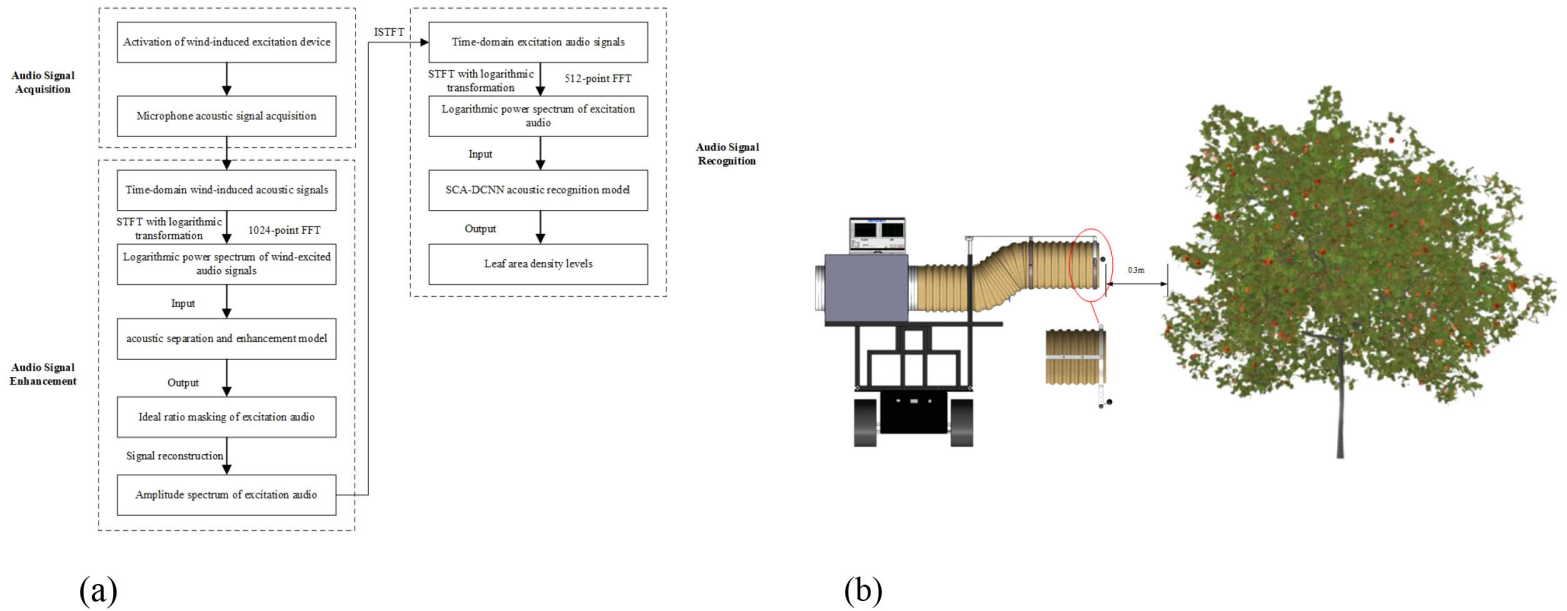
Workflow of leaf area density detection and schematic of audio signal acquisition scheme. **(a)** Workflow of leaf area density detection **(b)** Schematic diagram of audio signal acquisition.

Based on the leaf area density classification model previously developed by the research team (Li et al., 2024c), the leaf area density detection system in this study consists of three sequential stages: audio acquisition, audio enhancement, and audio-based recognition (**Figure 3a**).

Step 1: audio acquisition

Wind-induced canopy vibration was generated using a centrifugal fan operating at a wind speed of 10 m/s. A high-sensitivity PM420 microphone was positioned 0.3 m from the canopy. After the wind excitation device stabilized (approximately 5 s), time-domain excitation audio signals at each sampling unit were synchronously recorded using an audio acquisition device for a duration of 30 s (**Figure 3b**).

Step 2: audio enhancement

The audio signal was first processed using short-time Fourier transform (STFT) with an fast fourier transform (FFT) size of 1024, a frame shift of 512, and a Hamming window to extract the complex spectrogram. A logarithmic transformation was then applied to obtain the log-power spectrum (LPS). Each LPS frame was concatenated with three preceding and three following frames, resulting in a contextual feature vector with a total dimension of 7 × 513 = 3591. This vector was subsequently subjected to single-channel audio separation processing to derive the ideal ratio masks (IRM) corresponding to the excitation audio and background noise. Based on these masks, spectral masking was performed to enhance the original signal, which was then reconstructed into the time domain via inverse STFT (ISTFT).

Step 3: audio-based recognition

The enhanced signal underwent a second STFT and was resampled into a 256 × 256 log power spectrogram. This spectrogram served as a single-channel input image to an improved spatial attention convolutional neural network (SCA-DCNN)(Li et al., 2024c). The model consists of six convolution–pooling modules, incorporating multiple spatial attention mechanisms to enhance the weighted representation of time–frequency features. The final fully connected layer outputs a six-dimensional probability vector representing the leaf area density levels, which are mapped to the label set [‘A’, ‘B’, ‘C’, ‘D’, ‘E’, ‘F’].

This three-stage cascaded architecture combined signal denoising and feature extraction. It enabled real-time and reliable leaf area density classification under complex field conditions and provided critical data support for subsequent grid-based prescription spraying operations.

#### 2.2.2 Prescription map generation method

The prescription map serves as the spatial reference for the precise control of variable-rate spraying. Its core elements include the grid size, the grid center coordinates, and the mapping of required spray volumes. Based on over four years of orchard data, including row spacing (3.0–5.0 m), plant spacing (2.0–3.0 m), canopy diameter (2.0–3.0 m), and the response characteristics of the electric ball valve, the grid size was ultimately set to 3 m × 3 m. This setting was selected to balance spatial resolution with actuator dynamic performance. The center coordinates of each grid are obtained in real time via RTK-GNSS and are subsequently converted into planar coordinates using UTM projection. The spray requirement level (*S_i_*) is determined according to the leaf area density classification results described in Section 2.2.1. The six classified levels (A–F) are mapped to corresponding spray requirement levels (1–6), thereby establishing a high-precision one-to-one correspondence between the leaf area density and the required spray volume.

**Figure 4:**
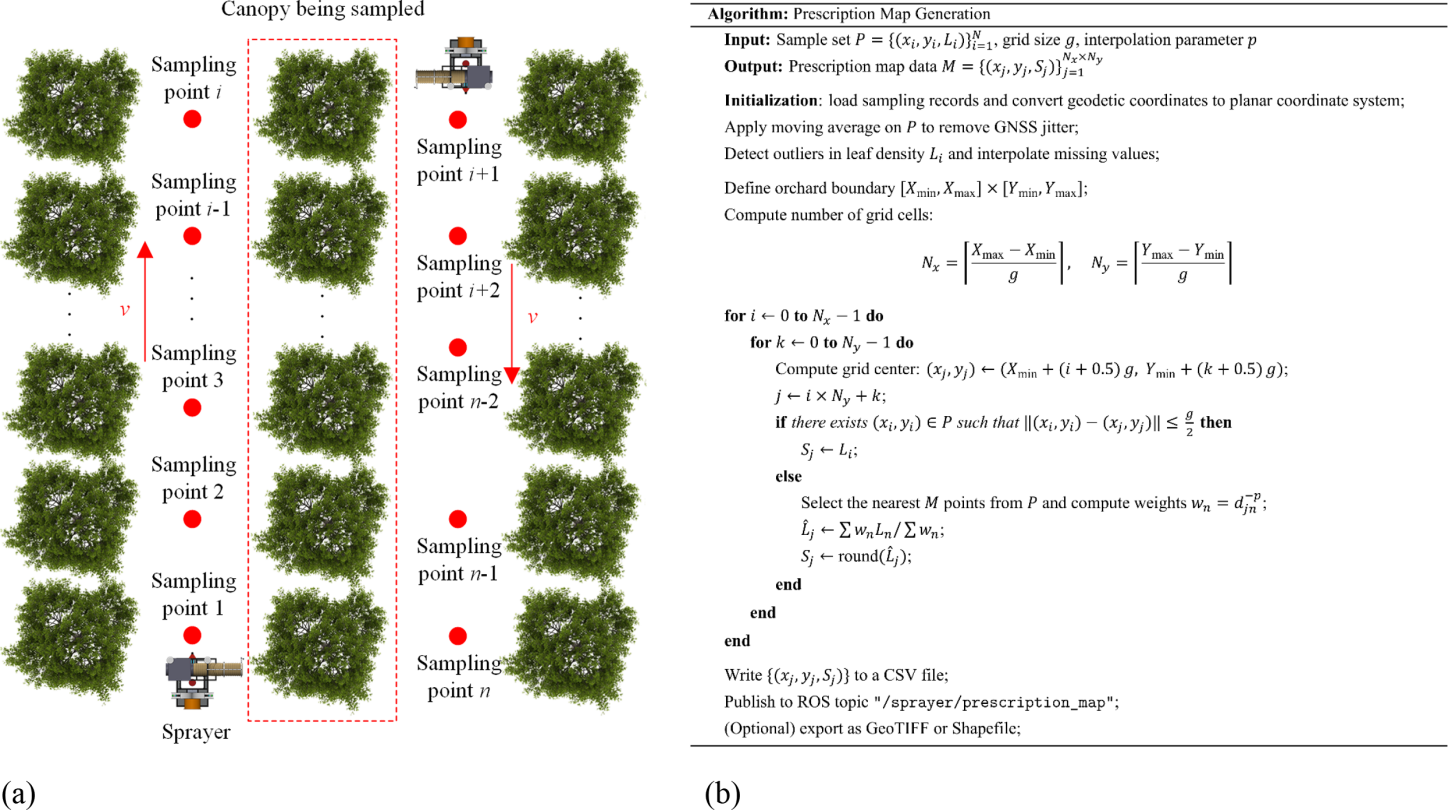
Methodology for prescription map generation. **(a)** GNSS positioning data acquisition and leaf area density estimation **(b)** Workflow for prescription map generation in orchards.

The prescription map generation process consists of two stages (**Figure 4**).

Step 1: global data acquisition

In remote-control mode, the spraying platform traverses the orchard rows at 3 m intervals. At each sampling point, the planar coordinates (*x_i_*, *y_i_*) and the corresponding leaf area density level (*L_i_*) are synchronously recorded. These data form a spatial distribution dataset of leaf area density covering the entire orchard.

Step 2: prescription map construction

Based on the established mapping relationship, the leaf area density level (*L_i_*) at each sampling point is converted into a spray requirement level (*S_i_*). All coordinate and spray requirement triplets (*x_i_*, *y_i_*, *S_i_*) are then sequentially structured and stored in a TXT file, generating standardized prescription map data.

This method features a compact structure and high operational efficiency. It supports dynamic updates and batch deployment, providing precise spatial decision-making references for subsequent path planning and closed-loop spraying operations.

### 2.3 Variable-rate spraying control method based on orchard-wide leaf area density distribution

#### 2.3.1 Construction method of the variable control model based on leaf area density

To satisfy the low-volume spraying criterion of achieving no fewer than 25 droplets per square centimeter on the leaf surface, a quantitative relationship was formulated between the canopy leaf area density and the required spray volume. This relationship is expressed in Equation 3 (Jiang et al., 2025):


(3) $$Q=\frac{5\times 10^{5}\times k_{q}\rho_{leaf}\pi C_{v}^{3}}{6\left(1-\delta_{b}L_{d}\right)P_{b}}$$

where *Q* is the required spray volume (L); *k_q_* is a correction coefficient (set to 3.3 in this study); *ρ_leaf_* is the leaf area density (m²·m⁻³); *C_v_* is a constant related to the nozzle, considering factors such as liquid surface tension and air density (set to 3.0 × 10⁻^4^ in this study); *L_d_* is the distance between the nozzle and the canopy (m); *δ_b_* is a correction coefficient accounting for spray losses (set to 0.1); and *P_b_* is the spray pressure (MPa).

This equation provides the theoretical foundation for linking spatially variable canopy structure to the spray dosage needed in precision orchard operations.

#### 2.3.2 Variable-rate spraying algorithm

**Figure 5:**
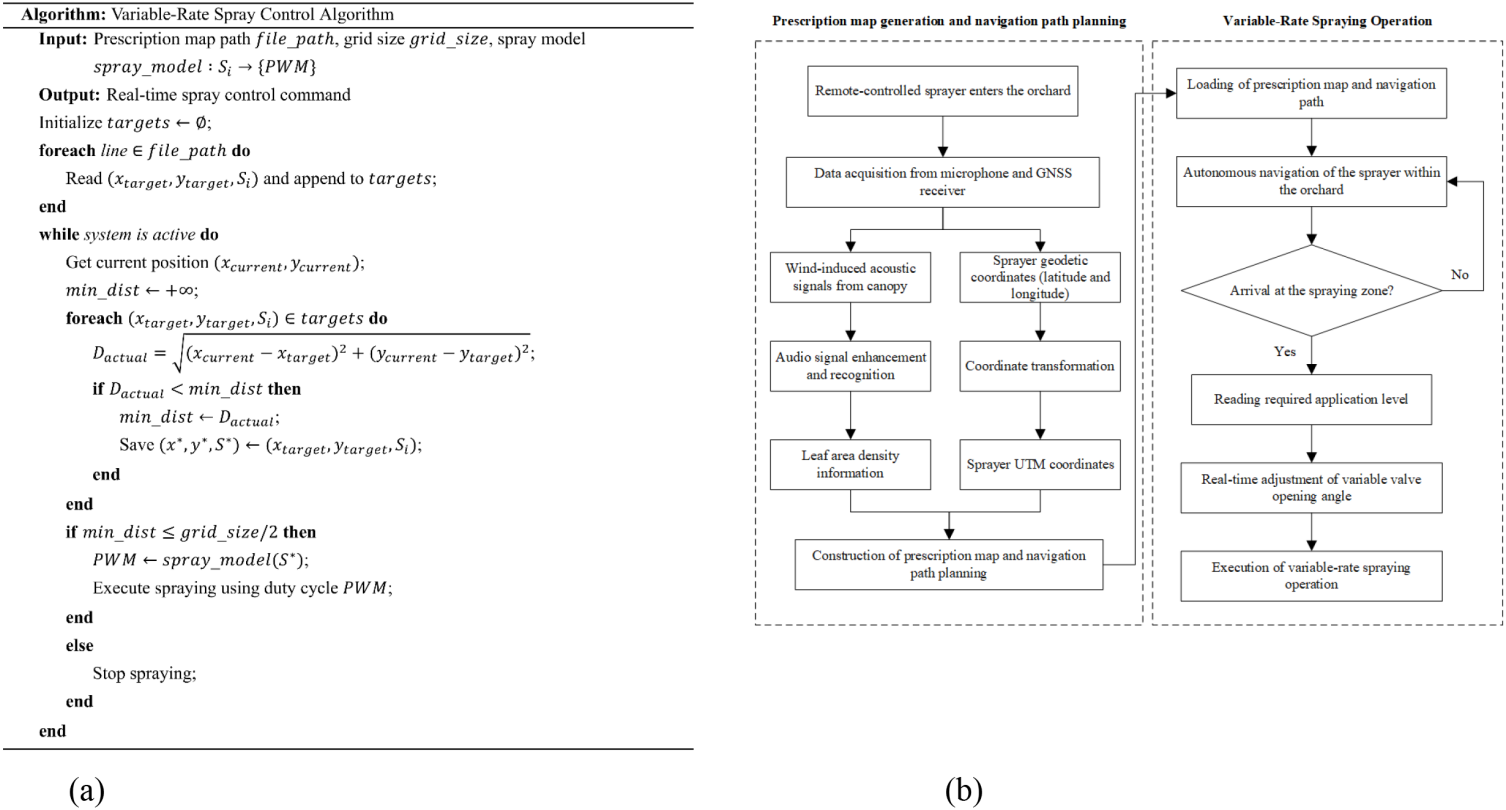
Variable-rate spraying control method based on orchard-wide leaf area density distribution. **(a)** Variable-rate spraying control algorithm **(b)** Workflow of the sprayer.

A fitting function *q = f(x_p_)* between the PWM duty cycle (*x_p_*) and the spray flow rate (*q*) was established through calibration experiments. Combined with the prescription map grid size of 3 m and an operational speed of 0.5 m/s, a closed-loop variable-rate spraying control model was constructed. The control algorithm was designed based on a four-layer architecture of “Data–Perception–Decision–Execution” (**Figure 5a**). The specific workflow is described as follows.

Step 1: data layer

The prescription map TXT file is imported, extracting the center coordinates *x_target_*, *y_target_*) of each grid along with the corresponding spray requirement level *S_i_*, which are stored as a data array(*x_target_*, *y_target_*, *S_i_*).

Step 2: perception layer

The current position of the sprayer (*x_current_*, *y_current_*) is obtained in real time via RTK-GNSS. The Euclidean distance between the current position and each grid center is calculated using Equation 4:


(4) $$D_{actual}=\sqrt{{\left(x_{current}-x_{target}\right)}^{2}+{\left(y_{current}-y_{target}\right)}^{2}}$$

Step 3: decision layer

When the actual distance D_actual_ is less than half of the predefined grid size (3 m), the system determines that the target grid has been reached and reads the corresponding spray requirement level (*S_i_)*.

Step 4: execution layer

Based on the spray requirement level *S_i_*, the spray volume is calculated using the model in Equation (3) and the calibration curve *q = f(x_p_)* to determine the required duty cycle (*x_p_*). A PWM command is then issued via the CAN bus to the lower controller to drive the variable-rate valve, achieving precise flow rate control.

This control method enables real-time optimization of the spray volume for each grid, significantly improving pesticide utilization efficiency and spray uniformity.

#### 2.3.3 Workflow of the variable-rate spraying system

As shown in **Figure 5b**, the operational workflow adopts an asynchronous architecture of “sensing–decision–actuation” and consists of three main stages:

1. Static multimodal sensing: After the sprayer is remotely driven into the target inter-row space and comes to a stationary position, the wind-excited device and the microphone synchronously collect canopy audio spectra and RTK-GNSS data at predefined grid intervals. The collected audio data were processed through the Audio Enhancement and Audio-Based Recognition stages, resulting in the classification of the corresponding leaf area density. The corresponding grid coordinates are determined through UTM projection. The classification results and positional data are saved as separate text files.
2. Dynamic decision-making optimization: Based on the leaf area density classification and coordinate data of each grid, the required spray level is computed. A point-based prescription map is then generated, and the optimal spraying path is fitted accordingly.
3. Precision spraying control: The system switches to autonomous navigation mode. The industrial computer (upper controller) continuously aligns the vehicle position with the corresponding prescription grid in real time. The lower controller dynamically adjusts the PWM duty cycle according to the spray volume required for each grid. This enables accurate control and ensures precision variable-rate spraying.

### 2.4 Orchard field testing and validation

#### 2.4.1 Experimental site: demonstration orchard

**Figure 6:**
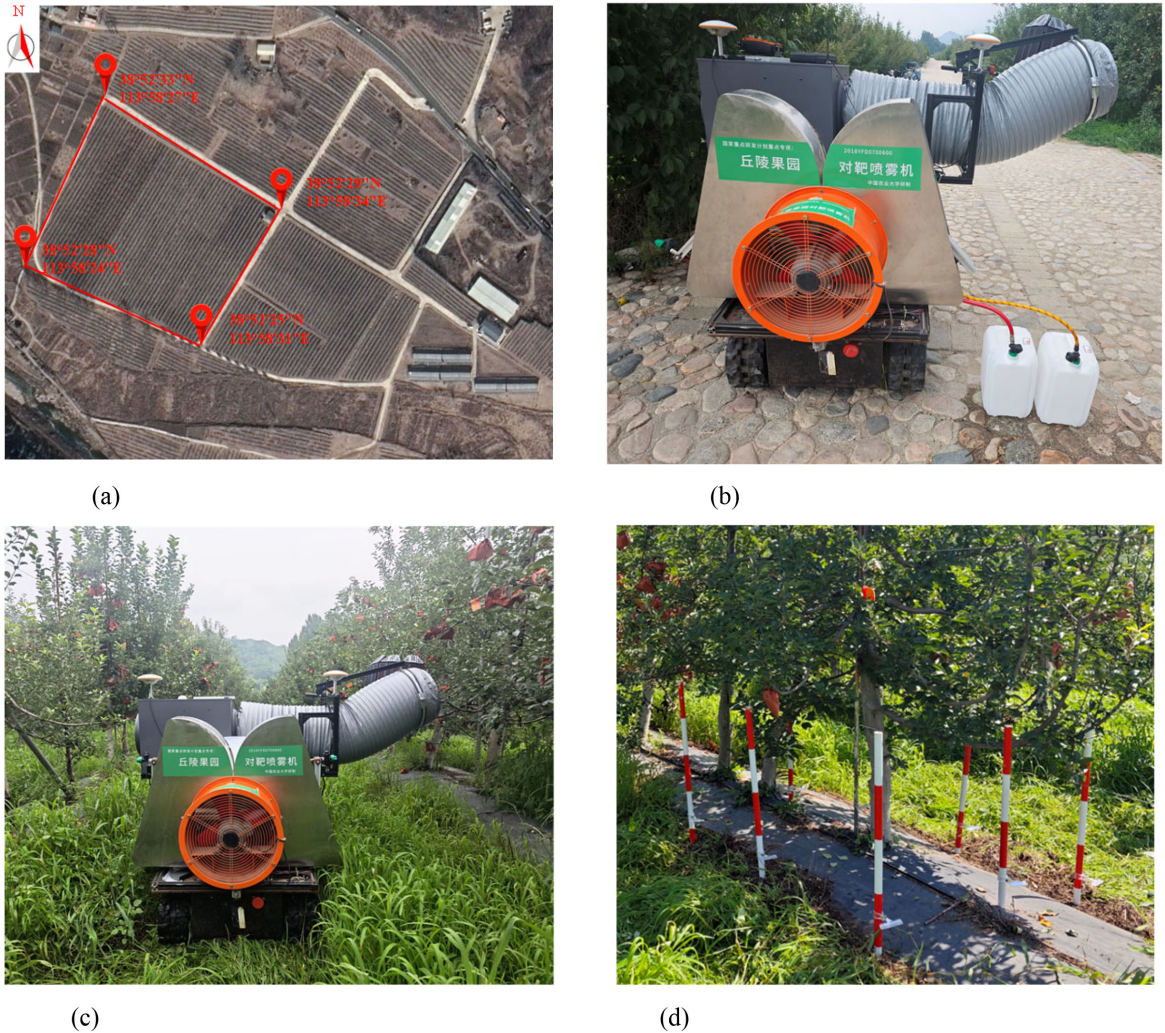
Experimental sites and test layout. **(a)** Location of the experimental site **(b)** Spray flow calibration test site **(c)** Field operation scene of the sprayer **(d)** Layout of the droplet deposition test site.

To enhance the representativeness and generalizability of this study, a standardized demonstration orchard was selected in Hongcaohe, Fuping County, Baoding City, Hebei Province, China (**Figure 6a**). The orchard featured a plant spacing of 2.0 m, a row spacing of 4.0 m, and an average tree height of 3.5 m. At this site, canopy leaf area density detection, wind-induced audio signal acquisition, spray flow rate calibration, and droplet deposition tests were conducted sequentially. The layout and field environment for each experiment are illustrated in **Figure 6**.

#### 2.4.2 Spray volume error analysis test

To verify the calibration accuracy and control performance of the variable-rate spraying system, spray volume calibration and precision testing were conducted in August 2024 (**Figure 6b**).

##### 2.4.2.1 PWM–flow rate calibration

In the single-side variable spraying mode, only the right-side valve was calibrated. The experimental setup included the self-propelled sprayer, a water bucket, an electronic scale, and a stopwatch. The calibration procedure was conducted as follows. After the sprayer pump was activated and stabilized, the nozzle was positioned over an empty water bucket. The system was operated sequentially at duty cycle settings ranging from 0% to 100% in 10% increments, with each setting maintained for 1 minute. After closing the valve, the mass of the liquid collected in the bucket was measured. Each duty cycle condition was tested three times, and the average value was calculated. If the relationship between the flow rate and the duty cycle exhibited nonlinearity, the duty cycle intervals were refined within the relevant ranges, and additional tests were performed to improve the calibration curve. The resulting fitting function *q = f(x_p_)* provided a quantitative basis for variable-rate spray control.

##### 2.4.2.2 Variable-rate spraying accuracy test

**Figure 7:**
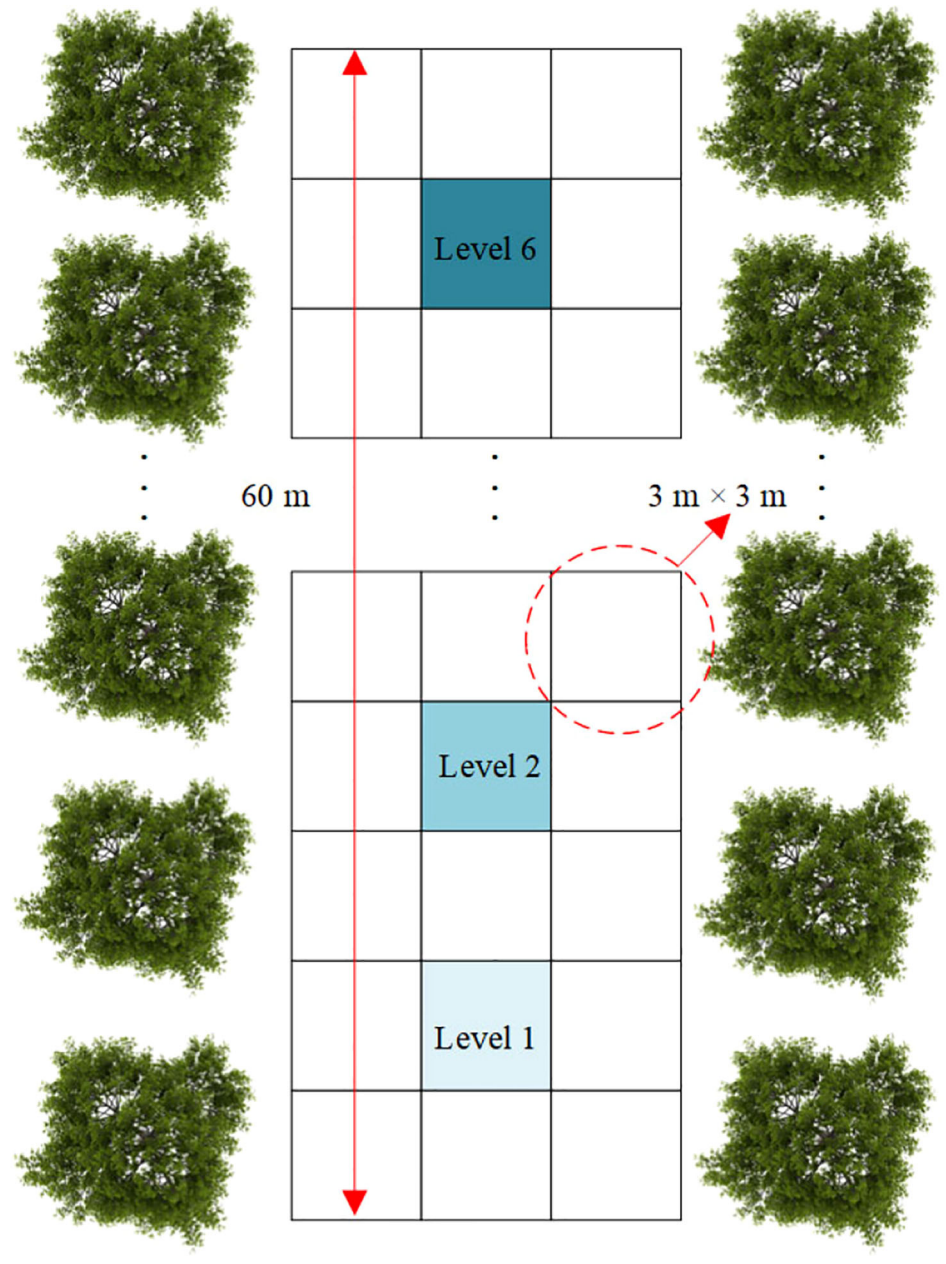
Control accuracy test scheme for variable-rate spraying.


In a 60 m long standardized testing area, the spraying operation zone was divided into six consecutive regions according to the spray requirement levels (Levels 1–6) specified in the prescription map. A buffer zone was established before and after each operational region to minimize transitional effects (**Figure 7**). The theoretical spray volume *Q’_i_* for each region was calculated based on the grid area and the corresponding prescription level. During the experiment, the nozzle was positioned over an empty water bucket, and spraying operations were sequentially performed for each spray requirement region. The actual spray volume (*Q_i_*) was measured by weighing the liquid collected in the bucket. Each region was tested three times to reduce random errors, and the mean value of the three measurements was used for analysis. The relative error between the actual and theoretical spray volumes for each region was calculated using Equation 5 to quantify the accuracy of the variable-rate spraying operation.

(5) $$\delta_{s}=\frac{\left|\bar{Q_{i}}-Q_{i}^{'}\right|}{Q_{i}^{'}}\times 100\%$$

This testing method effectively evaluates the stability and reliability of the variable-rate spraying algorithm and provides robust performance verification for the practical application of variable-rate spraying technology.

#### 2.4.3 Droplet deposition and runoff test

##### 2.4.3.1 Test location

To comprehensively evaluate the canopy coverage uniformity and ground runoff suppression performance of the variable-rate spraying system, comparative experiments between variable-rate and constant-rate spraying modes were conducted in August 2024 at the demonstration orchard.

The experiments were performed in accordance with the Chinese national standards NY/T 992–2006 *Air-assisted Orchard Sprayer Operation Quality* and JB/T 9782–2014 *General Test Methods for Plant Protection Machinery* (**Figures 6c, d**).

##### 2.4.3.2 Test design

**Figure 8:**
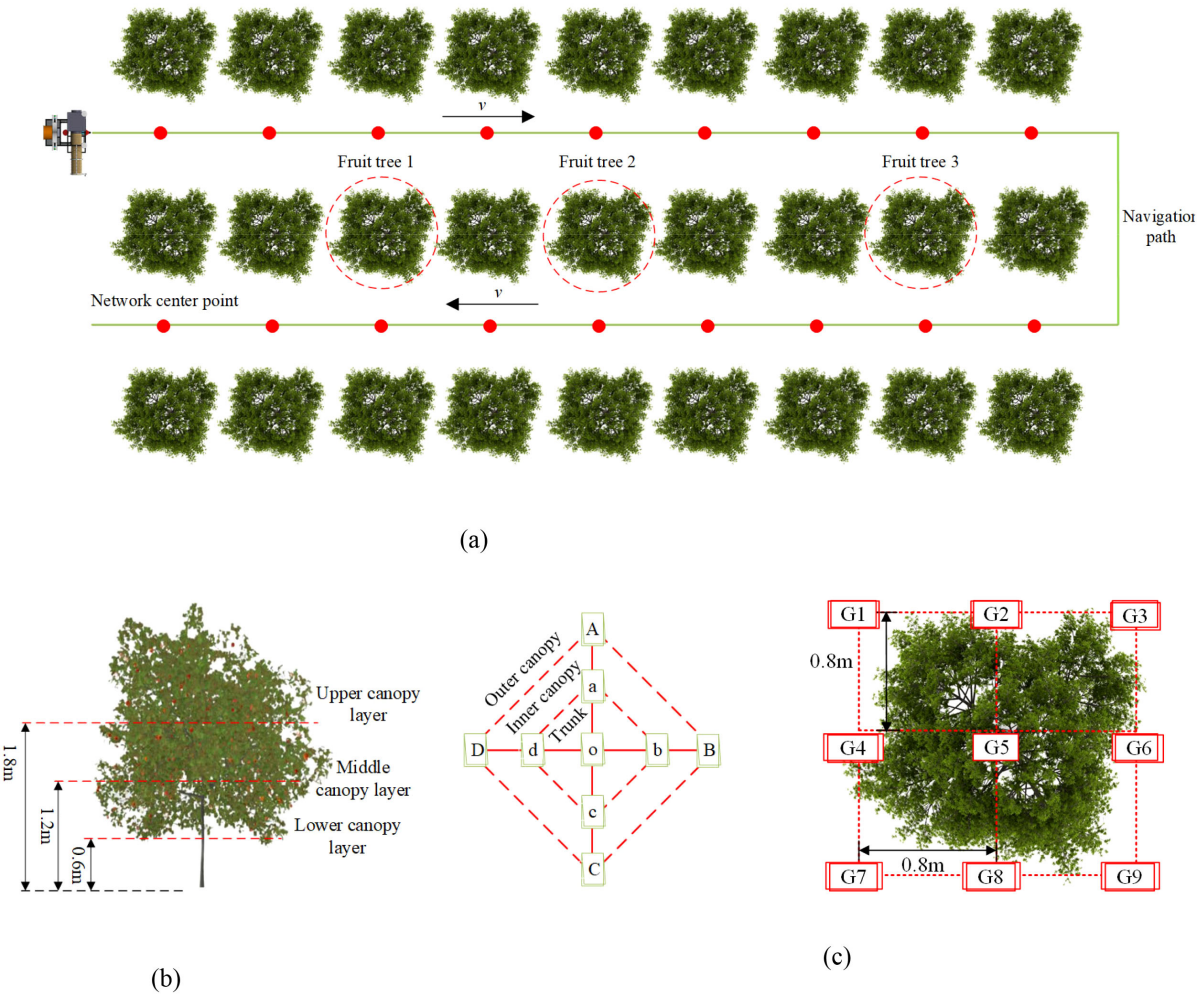
Experimental scheme for droplet deposition testing. **(a)** Sprayer operation scheme **(b)** Sampling point layout within the canopy **(c)** Ground-level sampling point layout.

The specific implementation of the droplet deposition test is illustrated in **Figure 8**. Three representative apple trees, labeled Tree 1, Tree 2, and Tree 3 (**Figure 8a**), were selected within the test area as sample trees. In the middle and lower canopy of each tree, three vertical testing layers were established at heights of 1.8 m (upper layer), 1.2 m (middle layer), and 0.6 m (lower layer). Each testing layer was further divided horizontally into nine sampling positions, including four outer positions (A, B, C, D), four inner positions (a, b, c, d), and one central trunk position (o). At each sampling position, water-sensitive papers were affixed to both the adaxial and abaxial surfaces of leaves using paper clips (**Figure 8b**).

To assess ground runoff, nine ground sampling points, labeled G1 to G9, were evenly distributed beneath the vertical projection of the canopy (**Figure 8c**). Water-sensitive papers were attached to ground marker rods using bulldog clips. Both variable-rate and constant-rate spraying tests were conducted under the same operational conditions. The driving speed was maintained at 0.5 m/s, the spraying pressure was set to 0.5 MPa, and water was used as the spraying medium. In the variable-rate spraying group, valve openings were dynamically adjusted according to the prescription map. In contrast, in the constant-rate spraying group, the valves remained fully open during operation.

##### 2.4.3.3 Data analysis method

After the experiments, all water-sensitive papers were collected and analyzed using DepositScan™ software, developed by the Chinese Ministry of Agriculture. The software was used to extract the droplet deposition volume per unit area (μL/cm²) and the droplet density (number/cm²). To minimize individual variability, the data at each sampling position were averaged across the three sampled trees. During data analysis, the droplet deposition count, droplet deposition volume, and the coefficient of variation (CV) of deposition volume were calculated for each sampling position. These indicators were used to comprehensively evaluate the uniformity and effectiveness of the spraying operation. Specifically, the droplet deposition count was used to assess whether the deposition met the minimum application requirement (≥25 droplets/cm²). The droplet deposition volume and its CV were used to quantify the uniformity of droplet distribution. Furthermore, the canopy droplet deposition characteristics were analyzed in two dimensions: longitudinally (across different canopy height layers) and transversely (across the outer, inner, and trunk regions).

The specific calculation methods are described as follows.

The droplet deposition volumes at the nine sampling positions in the upper, middle, and lower canopy layers are denoted as *X*_1_ *_j_*, *X*_2_ *_j_*, and *X*_3_ *_j_*, respectively, where j = 1, 2,…, 9. The longitudinal coefficient of variation (CV) of droplet deposition was calculated according to Equations 6–9, following the steps below:

Step 1: Calculate the average droplet deposition volume for each canopy layer.


(6) $$\{\begin{array}{c} {\bar{X}}_{u}=\frac{1}{9}\sum_{j=1}^{9}X_{1j}\\ {\bar{X}}_{m}=\frac{1}{9}\sum_{j=1}^{9}X_{2j}\\ {\bar{X}}_{l}=\frac{1}{9}\sum_{j=1}^{9}X_{3j} \end{array}$$

In the equations, ${\bar{X}}_{u}$ , ${\bar{X}}_{m}$ , and ${\bar{X}}_{l}$ represent the average droplet deposition volumes for the upper, middle, and lower canopy layers, respectively.

Step 2: Calculate the overall mean droplet deposition volume across the three canopy layers, denoted as *μ_v_*.


(7) $$\mu_{v}=\frac{{\bar{X}}_{u}+{\bar{X}}_{m}+{\bar{X}}_{l}}{3}$$

Step 3: Calculate the standard deviation of droplet deposition volume across the three canopy layers, denoted as *σ_v_*.


(8) $$\sigma_{v}=\sqrt{\frac{{\left({\bar{X}}_{u}-\mu_{v}\right)}^{2}+{\left({\bar{X}}_{m}-\mu_{v}\right)}^{2}+{\left({\bar{X}}_{l}-\mu_{v}\right)}^{2}}{3}}$$

Step 4: Calculate the longitudinal coefficient of variation of droplet deposition volume, denoted as *C_v_*.


(9) $$C_{v}=\frac{\sigma_{v}}{\mu_{v}}\times 100\%$$

where *C_v_* is the longitudinal coefficient of variation; *μ_v_* is the overall mean droplet deposition volume across the three canopy layers; and *σ_v_* is the standard deviation of droplet deposition volume across the three canopy layers.

The transverse coefficient of variation was calculated using Equations 10–13.


(10) $${\bar{X}}_{j}=\frac{X_{1j}+X_{2j}+X_{3j}}{9},\;j=1,2,\ldots ,9$$

(11) $$\mu_{h}=\frac{1}{9}\sum_{j=1}^{9}{\bar{X}}_{j}$$

(12) $$\sigma_{h}=\sqrt{\frac{1}{9}\sum_{j=1}^{9}{\left({\bar{X}}_{j}-\mu_{h}\right)}^{2}}$$

(13) $$C_{h}=\frac{\sigma_{h}}{\mu_{h}}\times 100\%$$

where *C_h_* is the transverse coefficient of variation; ${\bar{X}}_{j}$ is the mean droplet deposition volume across the three canopy layers at a single sampling point; *μ_h_* is the overall mean droplet deposition volume across the nine sampling points; and *σ_h_* is the standard deviation of droplet deposition volume across the nine sampling points.

This methodology systematically and quantitatively revealed the differences in canopy coverage uniformity and ground runoff between the variable-rate and constant-rate spraying modes. It provided a solid foundation for the performance validation and optimization of precision spraying technologies.

## 3 Results and discussion

### 3.1 Spray volume error analysis test results

#### 3.1.1 Experimental calibration results of spray flow rate in response to PWM duty cycle

**Figure 9:**
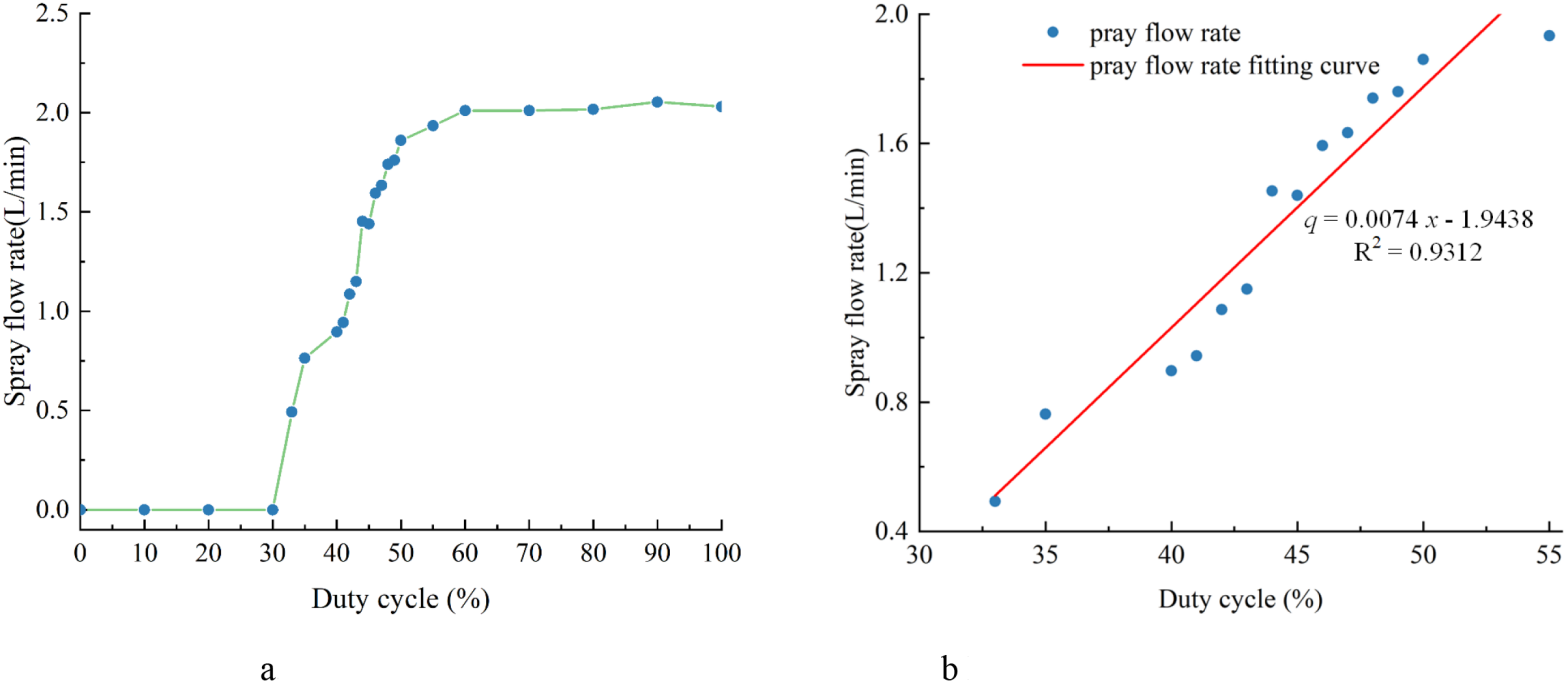
PWM–spray flow rate calibration results. **(a)** Experimental data **(b)** Duty cycle–spray flow rate fitting curve.


**Figure 9a** presents the calibration results between the PWM duty cycle (*x_p_*) and the spray flow rate (*q*), based on a total of 23 collected data sets. Analysis of the calibration data revealed the following patterns. When *x_p_* was less than 33%, the spray flow rate (*q*) remained approximately zero. When *x_p_* ranged from 33% to 55%, q increased approximately linearly with *x_p_*. When *x_p_* exceeded 55%, *q* tended to saturate. Therefore, as shown in **Figure 9b**, the data within the range of *x_p_* from 33% to 55% were used to fit the functional relationship between the spray flow rate (*q*) and the PWM duty cycle (*x_p_*), as expressed in Equation 14.

(14) $$q=0.0744x_{p}-1.9438\;(33\leq x\leq 55)$$

#### 3.1.2 Variable-rate spraying control model

Based on the operational parameters of a driving speed of 0.5 m/s and a prescription map grid size of 3 m × 3 m, a variable-rate spraying control model was established to translate spatially distributed canopy leaf area density levels into quantitative spray control signals. The model defines a one-to-one mapping between leaf area density levels, the corresponding spray requirement levels, the required spray volume per grid, and the PWM duty cycle applied to the electric spray valve.

The leaf area density was classified into six discrete levels (A–F), based on the field survey results described in Section 2.2. Each level reflects a distinct canopy density category, with level A indicating sparse foliage and level F representing the densest canopy conditions. For each leaf area density level, the corresponding spray requirement level was assigned from 1 to 6. The required spray volume was determined using Equation 3, accounting for droplet deposition thresholds and nozzle characteristics. Subsequently, calibration experiments were conducted to establish the functional relationship between spray volume and PWM duty cycle. The PWM duty cycles corresponding to each spray requirement level were derived from the fitted flow rate curve Equation 14. The complete variable-rate spraying control model is summarized in **Table 2**. This model enables real-time, grid-based adjustment of spray output according to canopy structure variability, providing a practical basis for precise and efficient field operations.

**Table 2:** Variable-rate spraying control model.

Leaf area density level (L_i_)	Spray requirement level (*S_i_*)	Required spray volume (L)	PWM duty cycle (%)
A	1	0.06	34
B	2	0.09	38
C	3	0.11	41
D	4	0.14	44
E	5	0.16	48
F	6	0.18	51

#### 3.1.3 Prescription map integration

**Figure 10:**
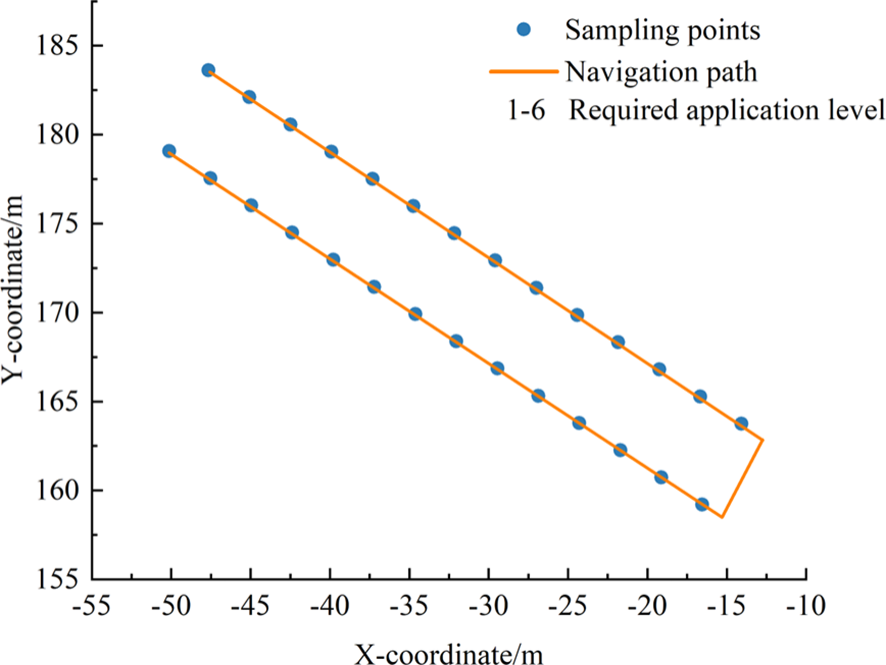
Orchard prescription map and sprayer navigation path.

Following the method described in Section 2.3, a prescription map with a grid size of 3 m × 3 m was constructed for three rows of apple trees within the test area. The navigation path was generated based on the center coordinates of each grid and the corresponding spray requirement levels (**Figure 10**). The prescription map and the navigation path were successfully imported into the sprayer’s control system. These data provided spatial decision-making references for the subsequent closed-loop spraying operations.

#### 3.1.4 Spray volume error analysis in field tests

The accuracy test results of variable-rate spraying are summarized in **Table 3**. The absolute relative errors between the average actual spray volume and the theoretical spray volume across different spray requirement levels ranged from 0.00% to 14.81%. The overall mean relative error was 5.52%. This result met the field accuracy requirement for orchard variable-rate spraying.

**Table 3:** Accuracy test results of variable-rate spraying.

Operation zone	Theoretical spray volume (L)	Actual spray volume (L)				Relative error (%)	Average relative error (%)
		Group 1	Group 2	Group 3	Mean Value		
1	0.06	0.06	0.06	0.06	0.06	0.00	5.52
2	0.09	0.08	0.07	0.08	0.08	14.81	
3	0.11	0.10	0.10	0.10	0.10	9.09	
4	0.14	0.15	0.15	0.15	0.15	7.14	
5	0.16	0.16	0.17	0.16	0.16	2.08	
6	0.18	0.18	0.18	0.18	0.18	0.00	

### 3.2 Droplet deposition and runoff test results

#### 3.2.1 Comparison of spraying distribution patterns

**Figure 11:**
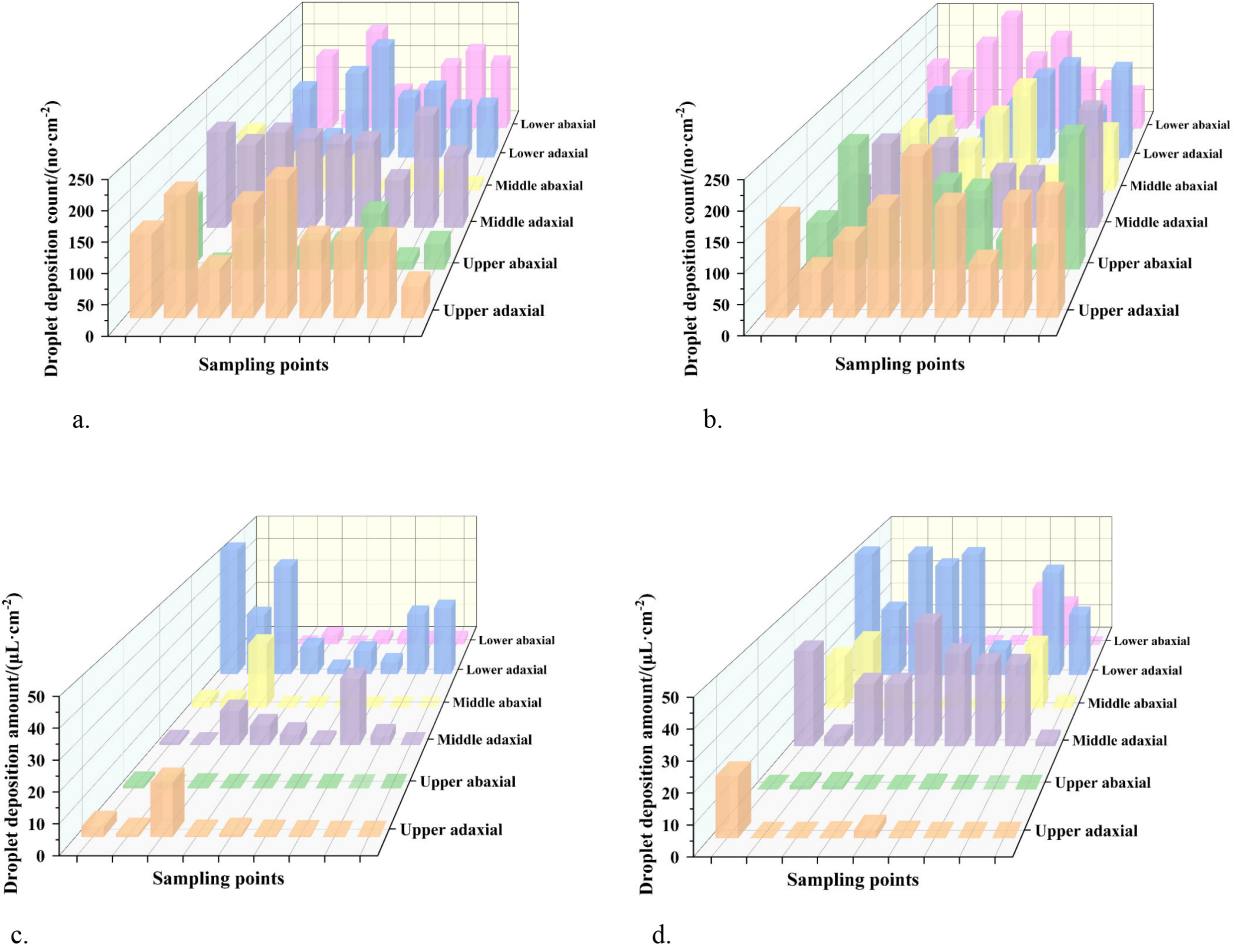
Canopy droplet deposition: count and amount distributions. **(a)** Variable-rate spraying - count distribution **(b)** Constant-rate spraying – count distribution **(c)** Variable-rate spraying – amount distribution **(d)** Constant-rate spraying – amount distribution.

The canopy droplet deposition patterns under variable-rate and constant-rate spraying modes are shown in **Figure 11**. Both spraying modes met the minimum application requirement of 25 droplets/cm². However, the variable-rate spraying mode significantly reduced pesticide usage compared to the constant-rate spraying mode.

#### 3.2.2 Analysis of longitudinal droplet deposition distribution characteristics

**Figure 12:**
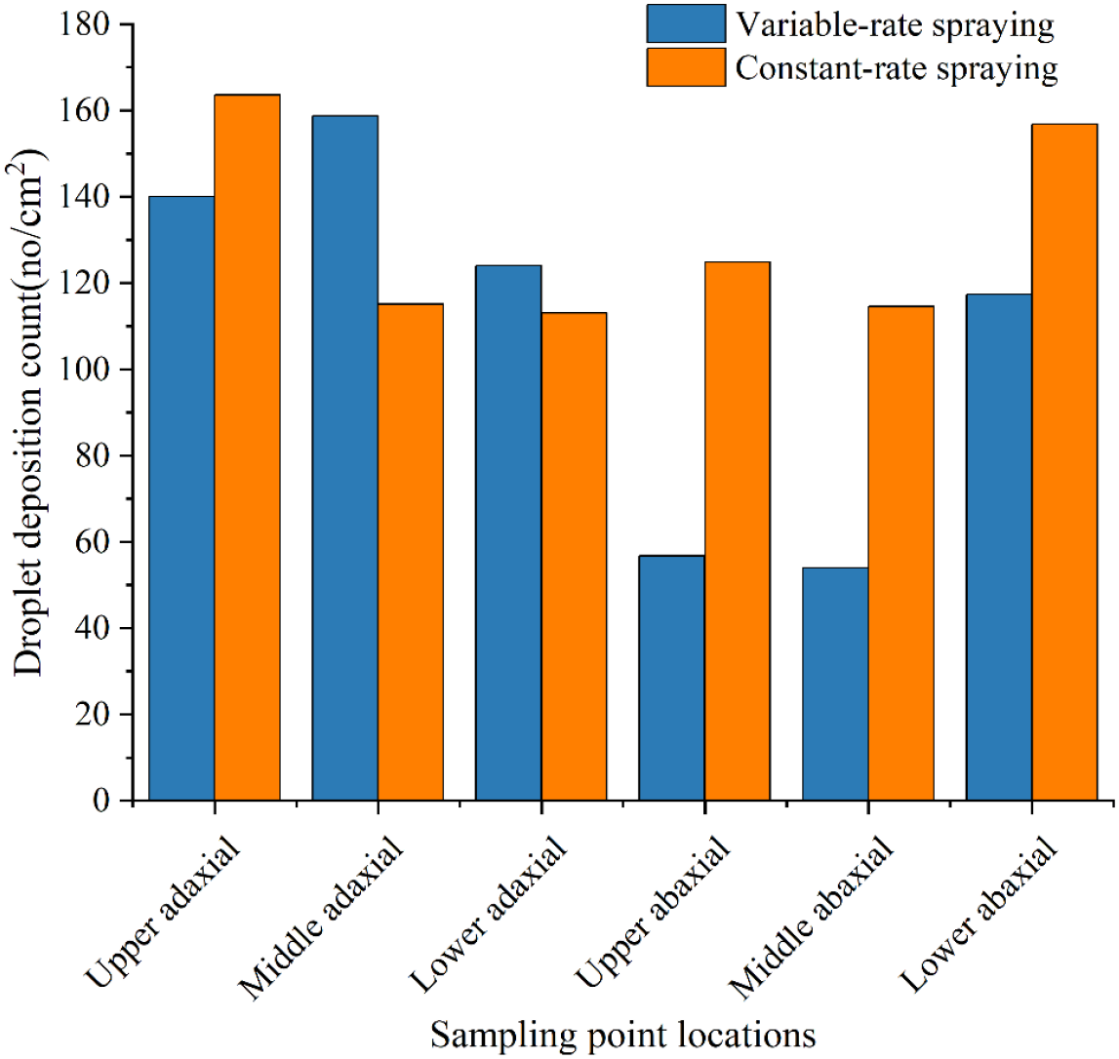
Average droplet deposition count distribution on adaxial and abaxial leaf surfaces across canopy layers.

The average droplet deposition on the adaxial and abaxial leaf surfaces was measured across the upper, middle, and lower canopy layers. The corresponding longitudinal coefficients of variation (CV) were also calculated to evaluate deposition uniformity. The results are summarized in **Figure 12** and **Table 4**.

**Table 4:** Longitudinal coefficient of variation (CV) across canopy layers.

Spraying mode	Sampling position	Droplet deposition (μL/cm²)		Longitudinal coefficient of variation (CV, %)	
		Adaxial surface	Abaxial surface	Adaxial surface	Abaxial surface
Constant-rate spraying	Upper layer	2.65	0.36	62.23	70.32
	Middle layer	4.32	2.58		
	Lower layer	9.07	2.75		
Variable-rate spraying	Upper layer	2.72	0.18	6.48	37.10
	Middle layer	2.47	0.37		
	Lower layer	2.81	0.39		

Analysis of **Figure 12** and **Table 4** indicates that the variable-rate spraying mode not only met the canopy spraying requirements but also reduced pesticide usage and improved longitudinal droplet deposition uniformity within the canopy. The detailed results are presented as follows:

##### 3.2.2.1 Spray coverage requirement

Under both the constant-rate and variable-rate spraying modes, the average droplet deposition counts on the adaxial and abaxial surfaces of leaves in each canopy layer exceeded 25 droplets/cm². This meets the minimum application requirement. The lowest deposition count was observed on the abaxial surface of the middle canopy layer under the variable-rate spraying mode. The value was 54 droplets/cm², which still significantly exceeded the threshold.

##### 3.2.2.2 Reduction in pesticide usage

The variable-rate spraying mode effectively reduced the amount of pesticide applied while still meeting the application requirements. Under the constant-rate spraying mode, the average droplet deposition volumes in the upper, middle, and lower canopy layers were 103.87%, 243.18%, and 370.15% higher, respectively, than those under the variable-rate spraying mode.

##### 3.2.2.3 Improved longitudinal deposition uniformity

The variable-rate spraying mode significantly improved the longitudinal uniformity of droplet deposition. Under the constant-rate spraying mode, the longitudinal coefficients of variation (CV) for the adaxial and abaxial leaf surfaces were 62.23% and 70.32%, respectively. In contrast, under the variable-rate spraying mode, the corresponding CVs were reduced to 6.48% and 37.10%. This represents reductions of 55.75% and 33.22% for the adaxial and abaxial surfaces, respectively.

#### 3.2.3 Analysis of transverse droplet deposition distribution characteristics

**Figure 13:**
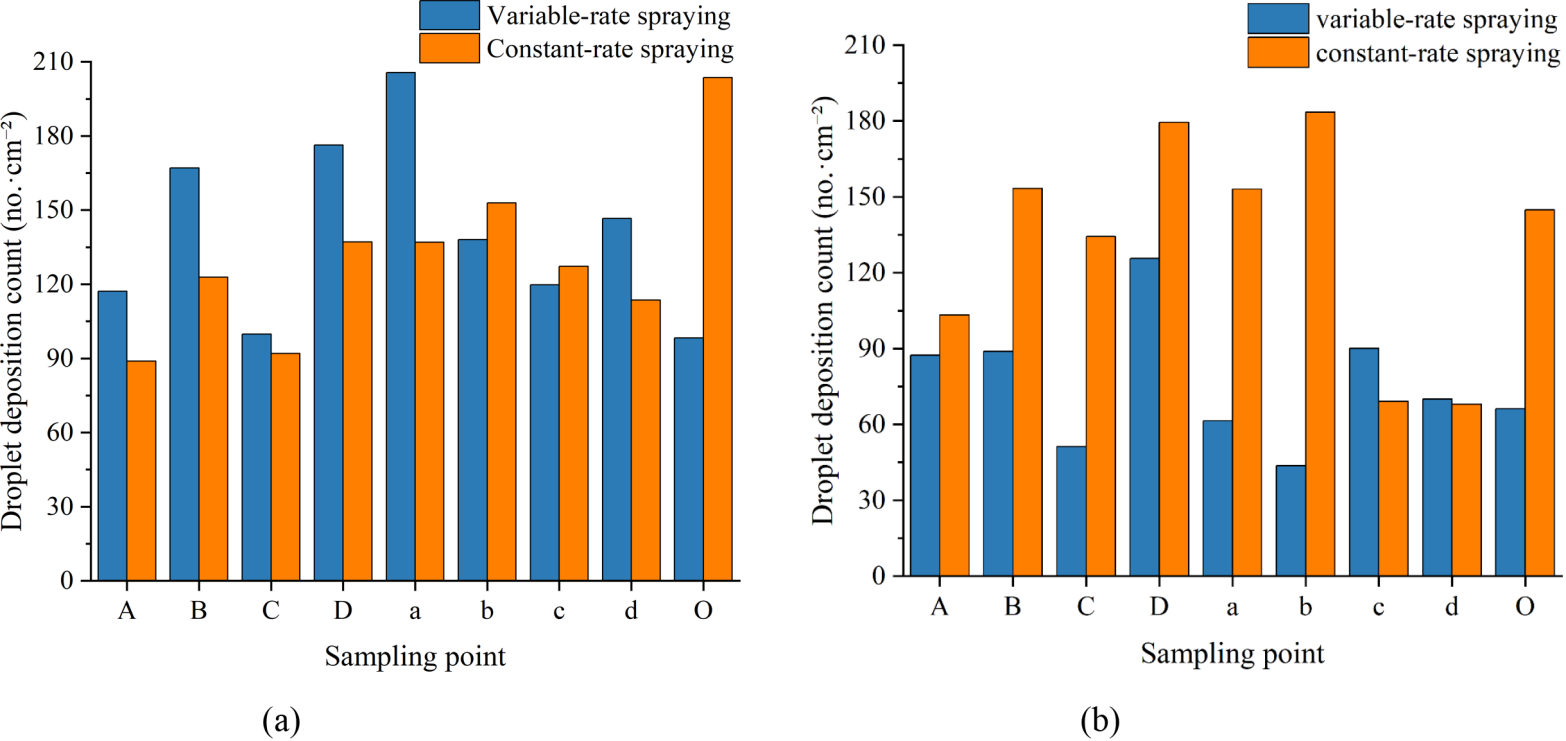
Distribution of average droplet deposition counts on adaxial and abaxial leaf surfaces at each sampling point. **(a)** Adaxial surface **(b)** Abaxial surface.

**Figure 13** shows the distribution of average droplet deposition counts on the adaxial and abaxial leaf surfaces at each sampling point. The results demonstrate that, under both spraying modes, the average droplet counts at all sampling points on both leaf surfaces exceeded 25 droplets/cm², thereby meeting the minimum application requirement. The lowest droplet count was recorded at the abaxial surface of sampling point *b* under the variable-rate spraying mode, with a value of 44 droplets/cm². Despite being the lowest, this value still significantly surpassed the threshold.

**Figure 14:**
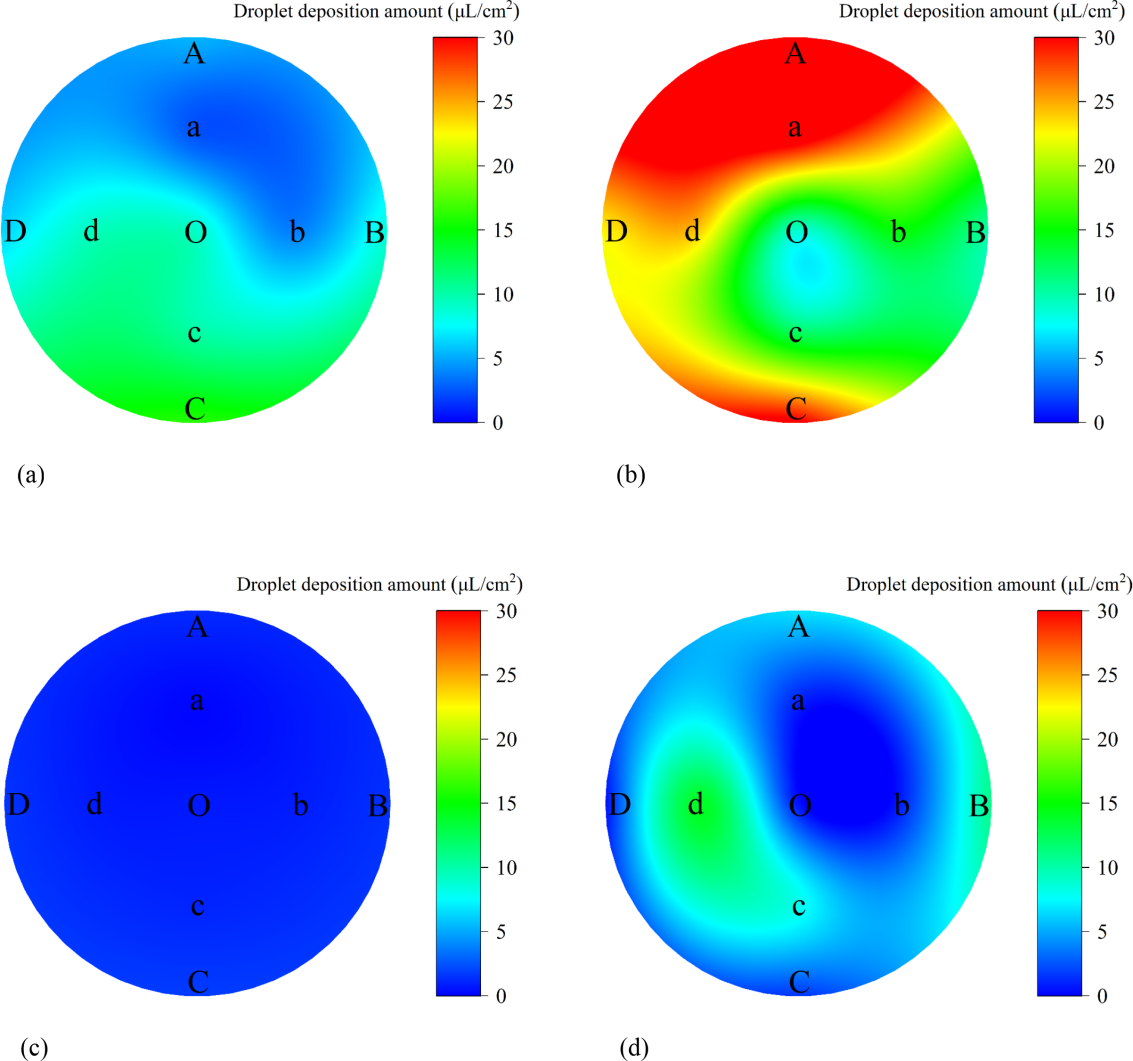
Distribution of average droplet deposition amounts on adaxial and abaxial surfaces across sampling points. **(a)** Adaxial surface under variable-rate spraying **(b)** Adaxial surface under constant-rate spraying **(c)** Abaxial surface under variable-rate spraying **(d)** Abaxial surface under constant-rate spraying.

The average droplet deposition volumes on the adaxial and abaxial leaf surfaces at each sampling point (**Figure 14**) and the canopy transverse coefficients of variation (CV) (**Table 5**) were analyzed to further investigate the uniformity of droplet deposition distribution in the transverse direction under both spraying modes. The results are summarized as follows:

1. Under constant-rate spraying, the transverse CVs of droplet deposition on the adaxial and abaxial leaf surfaces were 52.50% and 110.13%, respectively.
2. Under variable-rate spraying, the transverse CVs on the adaxial and abaxial surfaces were 51.82% and 47.81%, respectively.

**Table 5:** Transverse coefficient of variation (CV) within the canopy.

Spraying mode	Leaf surface	Coefficient of variation (CV)
Constant-rate spraying	Adaxial Surface	52.50%
	Abaxial Surface	110.13%
Variable-rate spraying	Adaxial Surface	51.82%
	Abaxial Surface	47.81%

These findings indicate that the variable-rate spraying mode effectively improved the uniformity of droplet deposition across sampling points on both leaf surfaces. In particular, the transverse CV on the abaxial surface was reduced by 62.32% compared to constant-rate spraying.

#### 3.2.4 Ground runoff analysis

**Figure 15:**
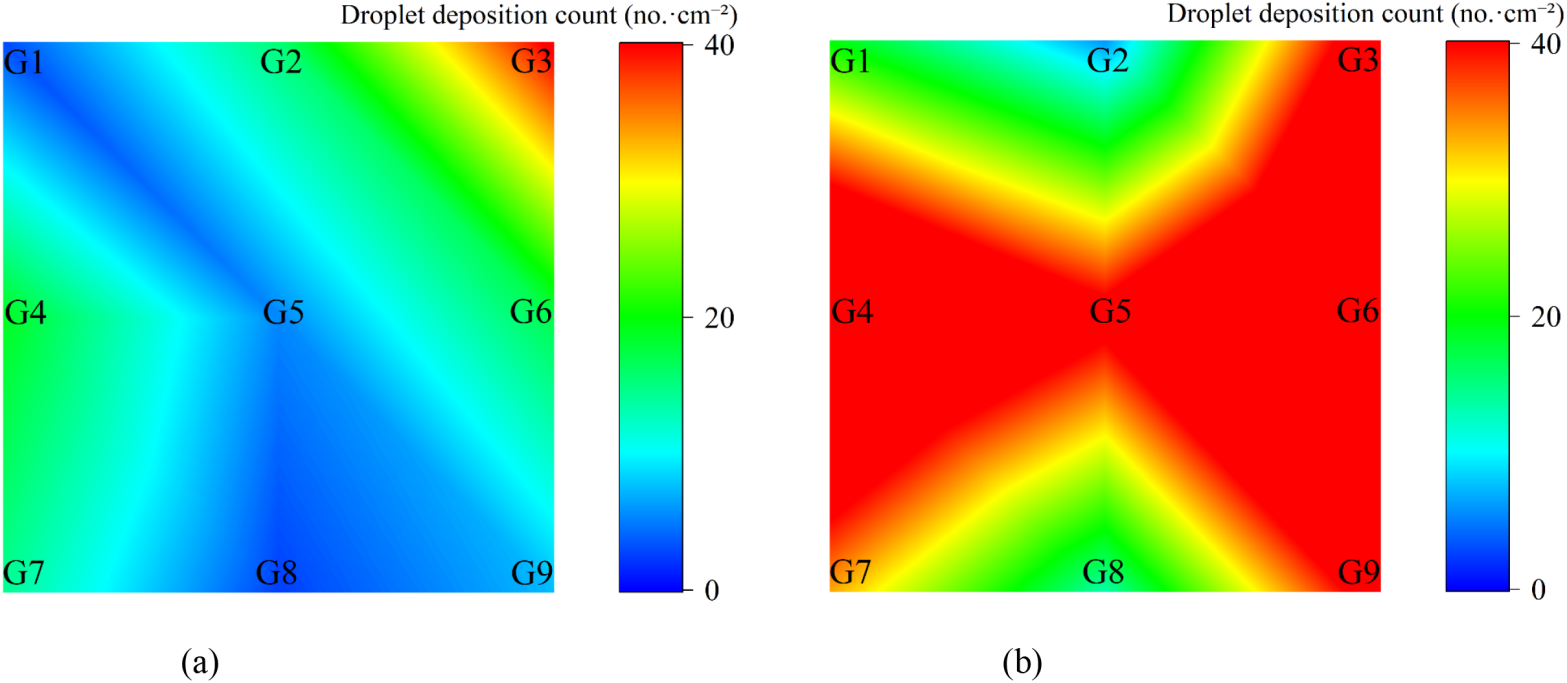
Distribution of ground runoff under variable-rate and constant-rate spraying. **(a)** Variable-rate spraying **(b)** Constant-rate spraying.

The ground runoff distribution under the two spraying modes is illustrated in **Figure 15**. It can be visually observed that the ground pesticide runoff under the variable-rate spraying mode was significantly lower than that under the constant-rate spraying mode.

The average ground pesticide runoff was 36.91 μL/cm² for the constant-rate spraying mode and 13.92 μL/cm² for the variable-rate spraying mode. Compared to constant-rate spraying, variable-rate spraying reduced ground runoff by approximately 62.29%.

### 3.3 Discussion

In this study, an integrated system combining audio signal analysis, canopy leaf area density classification, and variable-rate spray control was developed. The system incorporated wind-excited audio-conducted sensing with GNSS-based prescription mapping technologies. It effectively addressed the limitations of conventional sensors in detecting leaf area density within dense canopies. Moreover, it mitigated the decision-making delays commonly encountered in real-time variable-rate spraying systems. The proposed system also reduced hardware requirements for online computation and sensing. Its feasibility was fully validated through field experiments.

Compared with previous studies, most existing research employs ultrasonic sensors (Palleja and Landers, 2015), multispectral/red-green-blue (RGB) cameras (Tu et al., 2019; Raj et al., 2021), and LiDAR (Gu et al., 2022b) to detect canopy thickness, measure canopy height, and estimate canopy volume based on known travel speed. These methods generally assume that canopy foliage is uniformly distributed, thereby neglecting the internal heterogeneity of leaf area density. In contrast, the system proposed in this study is unaffected by audio diffraction and occlusion. It enables stable acquisition of excitation signals from deep within dense canopies, thereby facilitating the estimation of internal leaf area density. Additionally, sensor-based real-time variable-rate spraying systems typically rely on a single type of sensor (Zhang et al., 2019; Luo et al., 2024). These systems require real-time decoding and dynamic analysis of complex canopy features, which often results in low detection accuracy, slow response speeds, and limited decision-making capabilities. By integrating RTK-GNSS data with orchard-wide leaf area density distribution, a predefined prescription map was generated. This prescription map allows spray decisions to be executed simply by reading grid-based text files. The approach ensures high precision while significantly reducing reliance on onboard computational power. It demonstrates notable advantages in terms of cost efficiency, system deployment, and real-time performance.

Although the proposed system exhibits excellent performance, the current leaf area density estimation model is based on a limited set of samples from specific fruit tree varieties and growth stages. Therefore, further optimization is necessary through large-scale trials across multiple regions and crop types. Considering the system’s advantages of low cost, high robustness, and ease of deployment, it holds potential for extension to precision pesticide applications in modern orchards, tea plantations, and understory economic crops. In the future, the system could be enhanced by integrating meteorological sensors and multimodal data sources, such as vision and LiDAR. Environmental adaptation could be further improved through the application of attention mechanisms or graph neural networks. Furthermore, incorporating reinforcement learning-based online optimization algorithms may enable intelligent joint scheduling of valve opening degrees and operational paths. These advancements are expected to enhance resource utilization efficiency and environmental sustainability, thereby promoting the broader industrial application of this technology.

## 4 Conclusion

This study presents an orchard precision spraying system that integrates wind-excited audio-conducted sensing, grid-based prescription mapping, and variable-rate spray control. A high-precision mapping relationship among leaf area density, required spray rate, and PWM duty cycle was established. The system was validated through field experiments conducted in a commercial orchard. The main conclusions are as follows:

(1) Completion of prescription map generation and system integration: A 3 m × 3 m grid-based prescription map was successfully generated using RTK-GNSS-acquired operation trajectories and classified leaf area density data. A mapping curve linking leaf area density, required spray rate, and PWM duty cycle was established through calibration experiments, achieving a goodness of fit of R² = 0.93. The system integrated an electric tracked chassis, an autonomous navigation module, variable-rate spraying actuators, and a ROS-based control platform. It enabled online loading of the prescription map, path fitting, and coordinated control between the upper and lower computers for dynamic adjustment of the spray valves.

(2) Validation of spraying performance and operational benefits: The variable-rate spraying system satisfied the canopy spraying requirements while significantly reducing pesticide usage and improving spray uniformity. Experimental results showed that, under the variable-rate spraying mode, the longitudinal coefficients of variation (CV) for droplet deposition were 6.48% on the adaxial surface and 37.10% on the abaxial surface. Compared to constant-rate spraying, these values represented reductions of 55.75% and 33.22%, respectively. The transverse CVs were 51.82% on the adaxial surface and 47.81% on the abaxial surface, with reductions of 0.68% and 62.32%, respectively. Furthermore, the ground runoff of spray solution was reduced to 13.92 μL/cm², representing a 62.29% decrease compared to constant-rate spraying.

In conclusion, this study provides a complete technical system solution for orchard plant protection. The proposed methodology can be extended to similar orchard scenarios, such as pear and peach orchards. It offers practical value in enhancing spraying precision and reducing pesticide non-point source pollution.

## Data Availability

The original contributions presented in the study are included in the article/supplementary material. Further inquiries can be directed to the corresponding authors.
